# Cytochrome C-like Domain Within the Human BK Channel

**DOI:** 10.3390/ijms26157053

**Published:** 2025-07-22

**Authors:** Taleh Yusifov, Fidan Qudretova, Aysel Aliyeva

**Affiliations:** Institute of Biophysics, Ministry of Science and Education of the Republic of Azerbaijan, Z. Khalilov Street, 117, Baku AZ1141, Azerbaijan; fqudretova@gmail.com (F.Q.); taqiyevaaysel0@gmail.com (A.A.)

**Keywords:** MaxiK, Slo1, cytochrome c, heme regulatory motif, peroxidase activity

## Abstract

Large-conductance, voltage- and calcium-activated potassium (BK) channels are crucial regulators of cellular excitability, influenced by various signaling molecules, including heme. The BK channel contains a heme-sensitive motif located at the sequence *^612^CKACH^616^*, which is a conserved heme regulatory motif (HRM) found in the cytochrome c protein family. This motif is situated within a linker region of approximately 120 residues that connect the RCK1 and RCK2 domains, and it also includes terminal α-helices similar to those found in cytochrome c family proteins. However, much of this region has yet to be structurally defined. We conducted a sequence alignment of the BK linker region with mitochondrial cytochrome c and cytochrome c domains from various hemoproteins to better understand this functionally significant region. In addition to the HRM motif, we discovered that important structural and functional elements of cytochrome c proteins are conserved in the BK RCK1-RCK2 linker. Firstly, the part of the BK region that is resolved in available atomic structures shows similarities in secondary structural elements with cytochrome c domain proteins. Secondly, the Met80 residue in cytochrome c domains, which acts as the second axial ligand to the heme iron, aligns with the BK channel. Beyond its role in electron shuttling, cytochrome c domains exhibit various catalytic properties, including peroxidase activity—specifically, the oxidation of suitable substrates using peroxides. Our findings reveal that the linker region endows human BK channels with peroxidase activity, showing an apparent H_2_O_2_ affinity approximately 40-fold greater than that of mitochondrial cytochrome c under baseline conditions. This peroxidase activity was reduced when substitutions were made at *^612^CKACH^616^* and other relevant sites. These results indicate that the BK channel possesses a novel module similar to the cytochrome c domains of hemoproteins, which may give rise to unique physiological functions for these widespread ion channels.

## 1. Introduction

BK channels are K^+^-selective, voltage-, and Ca^2+^-dependent channels that regulate fundamental cellular functions [1,2,3,4]. Their role in controlling vascular tone is well-established. Their unitary K^+^ conductance (~250 pS), an order of magnitude larger than that of typical voltage-gated K^+^ channels, renders BK channels, powerful regulators of the cell membrane, potential and it is not surprising that serious human diseases have been associated with BK channel malfunction, including epilepsy and paroxysmal movement disorder [5,6].

**Figure 1:**
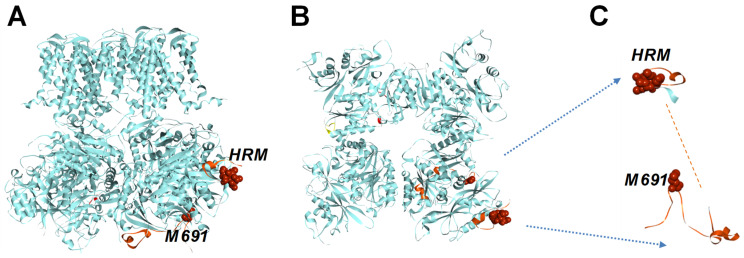
BK channel structure and position of functionally important residues for heme binding. Side view of a BK channel (**A**) and C-terminal gating ring (**B**) atomic structural (*PDB #6V3G*). The position of a conserved heme-regulatory motif (HRM, *^612^CKACH^616^*) and the putative second heme axial ligand M691 is shown. (**C**) Conserved heme coordination residues *^612^CKACH^616^* and Met691 are shown in the available atomic structure of the linker region between RCK1-RCK2 domains (*PDB #6V3G*).

While intracellular Ca^2+^ is typically recognized as the main regulator of BK channels, numerous other small signaling molecules, including Mg^2+^, H^+^, Ba^2+^, NO, CO, heme/hemin, and nutrients such as omega-3 fatty acids are capable of potently modulating the channel open probability [1,7,8,9,10,11,12,13,14]. The ligand-dependent gating of the BK channel is mediated by the gating ring (GR) apparatus—a large torus-shaped structure formed by the assembly of the C-termini of four BK channel α subunits (Figure 1A,B) [15,16,17,18,19].

Recent data have revealed a new role for the BK channel as a sensor of intracellular free hemin, the oxidized form of heme [Fe(II) protoporphyrin-IX]. Hemin [Fe(III) protoporphyrin-IX] facilitates the activation of BK channels at hyperpolarized potentials. However, it decreases the probability of channel opening at depolarized potentials [20,21,22]. Heme is an important cell-signaling molecule and influences the activity of other ion channels such as Kv1.4 [23], KATP [24], epithelial sodium channel [25], and ether à go-go (hEAG1, Kv10.1) [26] and Kv3.4 [27], but the physiological significance of heme-dependent modulation of ion channel activity remains unknown.

Several mechanisms have been proposed regarding the modulation of BK channel activity by heme. Heme may be involved in BK-dependent physiological processes such as oxygen sensing [28], which is related to heme’s sensitivity to a biologically signaling molecule such as CO [7,29]. Inhibition of BK channels by heme may also play a regulatory or compensatory role during vascular events such as stroke hemorrhage [20,21]. Future studies based on native BK channels will provide insights into the physiological role of heme regulatory function.

The BK channel ring is heme sensitive at the prosthetic group sitat site *^612^CKACH^616^* [22,29,30], which is compatible with the conserved heme-binding motif CXXCH among the cytochrome c protein family [31]. The heme-binding site lies a ~120-residue linker connecting two of the Ca^2+^-sensing modules, RCK1 and RCK2 domains (Figure 1A,B), which has so far evaded atomic-level resolution in BK structure studies due to its unordered structure (Figure 1C). In this work, we provide evidence that the BK channel heme-binding region shares a remarkable similarity with hemoprotein cytochrome c family proteins, one of the most extensively studied proteins. The mitochondrial cytochrome c is one of the first to be crystallized, and it is primarily known for its role in the respiratory chain in mitochondria. Aside from its well-recognized role in electron shuttling, cytochrome c also performs a catalytically, peroxidase activity [32]. Cytochrome c as well as other heme-bound peroxidases, can convert H_2_O_2_ into water in the presence of an electron donor. Based on this view, we have tested that the BK channel is more than the ligand-binding domain of the BK ion channel; it can also perform enzymatic functions under its heme-bearing cytochrome c-like domain. We hypothesized that, through their peroxidase activity, BK channels may perform multifunctional roles in cells.

## 2. Results

### 2.1. The Human BK Channel Structure Comprises a Cytochrome C-like Feature Within the Gating Ring

**Figure 2:**
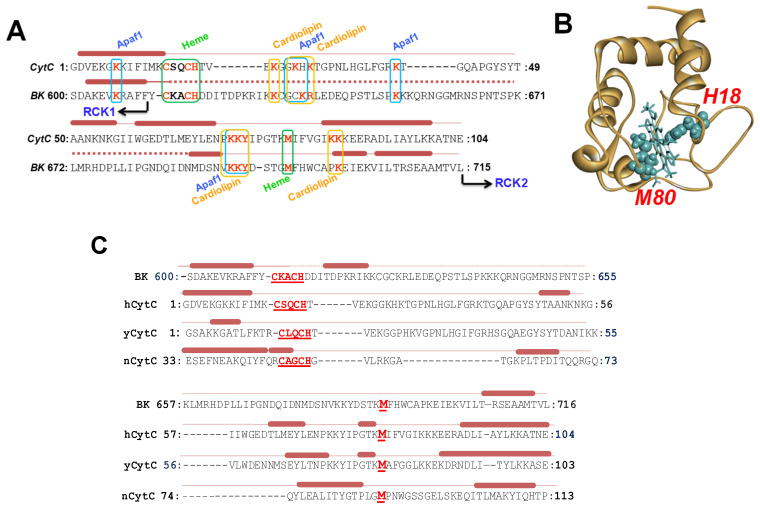
The primary sequence of the human RCK1-RCK2 linker region aligned with human cytochrome c (CytC) family proteins. (**A**) The primary sequence of human cytochrome c (CytC) aligned with the linker region of the human BK (BK). The secondary structures, obtained from *PDB #1J3S* for the human cytochrome c, and *PDB #6V3G* for the human BK channel C terminus, are shown above the sequences: bars: α helix; solid line: disordered; dotted line: unresolved. The functional residues of cytochrome c for interaction with cardiolipin (orange), Apaf1 (blue) and also residues for heme coordination (green: HRM and M80) are shown in boxes, above conserved residues in the BK sequence. (**B**) Three-dimensional structure of the human mitochondrial cytochrome c, Cyt C (*PDB ID: 1J3S*) [33]. Positions of axial ligands to the heme iron amino acids His18 and Met80 were shown in spheres. (**C**) Structure-based sequence alignment of the RCK1-RCK2 linker region (*^599^ASD*…*TVL^715^*, *PDB #6V3G*), and cytochrome c fold proteins: hCytC, human mitochondria cytochrome c (*PBD #1J3S*), yCytC, yeast iso-1-cytochrome c (*PDB #1YFC*) [34], nCytC, cytochrome c domain of the nitrite reductase from *Pseudomonas aeruginosa* (PDB #1NIR) [35]. The secondary structures are shown above the sequences: bars: α helix; solid line: disordered; dotted line: unresolved. Conserved heme coordination residues CXXHC and Met are shown in red. The sequences of the BK channel linker region and cytochrome c proteins are aligned CLUSTALW 2.1 computer program.

The linker region is between RCK1-RCK2 domains comprising *^612^CKACH^616^,* and it is compatible with the conserved heme-binding motif CXXCH among the cytochrome c protein family [31]. The heme-binding site lies a ~120-residue linker connecting two of the Ca^2+^ sensing modules, RCK1 and RCK2 domains (Figure 1A,B), which has so far evaded atomic-level resolution in BK structure studies (Figure 1C). To test the hypothesis that the BK gating ring comprises a cytochrome c-like structure, we performed a sequence alignment of the C-terminal portion of the RCK1 domain and the downstream RCK1-RCK2 linker (*^599^ASD*…*TVL^716^*) with human mitochondrial cytochrome C (*hCytC*, *PBD #1J3S*). We found that, in addition to the heme-regulated motif HRM (*^14^CSQCH^18^*), key structural elements of human mitochondrial CytC are conserved in this region of the human BK channel (*^612^CKACH^616^*) (Figure 2A): firstly, the portion of the BK region resolved in the available atomic structures shares secondary structure elements (N- and C-terminal α helices with CytC family proteins; secondly, CytC positively charged residues critical for Apaf-1 and cardiolipin interaction align with BK residues K606, K623, R648, K684, K685, and K698; moreover, CytC methionine residues position at 80, the second axial ligand to the heme iron atom aligns with BK channel M691 [32]. Cytochromes c is among the most studied proteins. The three-dimensional structure of mitochondrial cytochrome c was solved in 1970 [31] (Figure 2B).

**Figure 3:**
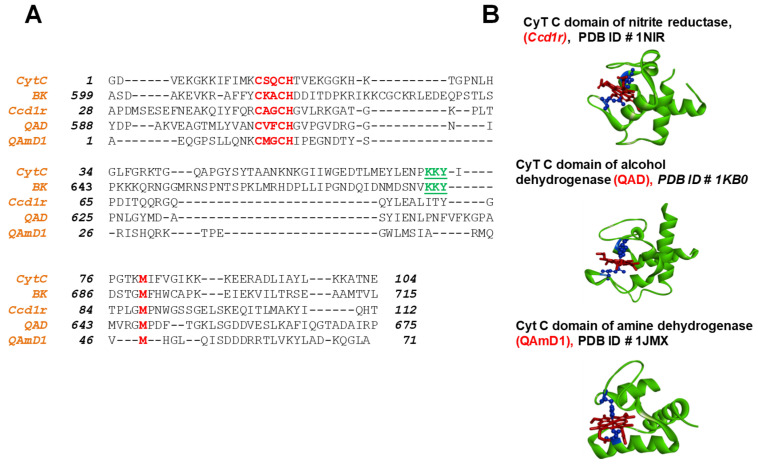
Sequence alignment of mitochondrial cytochrome c (CytC), human BK linker regions (BK) with cytochrome c domains from different organisms. (**A**) The sequences of the mitochondrial cytochrome c (*CytC*, *PDB #1J3S*), linker region of the human (*BK*, *PDB #6V3G*), cytochrome c domains from nitrite reductase, (*Ccd1r*, *PDB ID #1NIR*), of alcohol dehydrogenase (*QAD*, *PDB ID #1KB0*) [36] and amine dehydrogenase (*QAmD1*, *PDB ID #1JMX*) [37] were aligned using CLUSTALW. (**B**) The conserved HRM motif (*CKACH*) and methionine residues in the corresponding sequences are indicated in red. Results obtained from these analyses demonstrated that the linker region between RCK1-RCK2 domains share similar features with another cytochrome c family proteins; in addition to the HRM (*CXXCH*) motif, the second axial ligand to the heme iron Met residues, aligns with BK channel M69. Moreover, the linker region alpha-helices share Cytochrome C protein’s secondary structure elements (Figure 2C).

Cytochrome c binds to heme through two thioether bonds involving the sulfhydryl groups of two cysteine residues, specifically *C14* and *H18*. These cysteine residues can form a reversible thiol/disulfide redox switch that regulates the affinity of the BK C-terminal domain for heme. The heme iron ion is always axially coordinated by a H616. Cytochrome c proteins are found throughout all organisms and belong to the cytochrome c-fold superfamily. These cytochrome c-fold proteins share common features: they are predominantly composed of alpha-helices and exhibit structural similarities despite having poorly conserved primary sequences. However, the heme coordination motif and the methionine (Met) residues are conserved among them. We investigated the structure-based sequence alignment of the human BK channel C-terminus (BK, PDB #6V3G) with several members of the cytochrome c family: human mitochondrial cytochrome c (hCytC, PBD #1J3S), yeast iso-1-cytochrome c (yCytC, PDB #1YFC), and the cytochrome c domain of nitrite reductase from Pseudomonas aeruginosa (nCytC, PDB #1NIR) (Figure 2C). Additionally, we performed sequence analyses of cytochrome c domains from different proteins (Figure 3).

**Figure 4:**
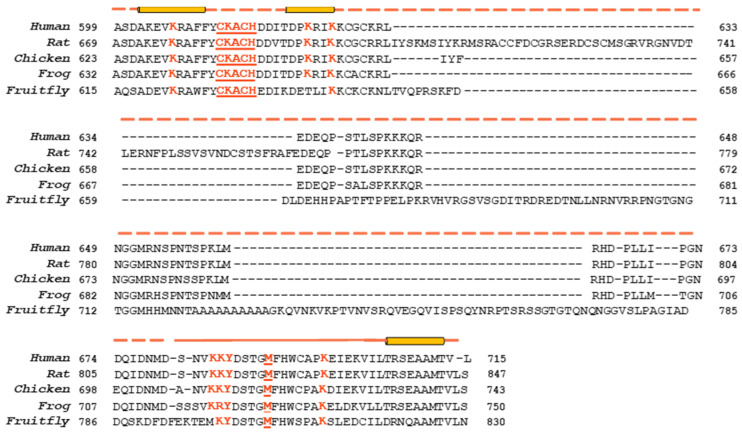
The sequence alignment of BK channel linker regions across different organisms. Partial sequences of BK (Slo1) channels from *Homo sapiens* (human), *Rattus norvegicus* (rat), *Gallus gallus* (chicken), *Xenopus laevis* (frog), *Drosophila melanogaster* (fruitfly), were aligned using CLUSTALW. The secondary structure (α-helices) of the human BK channel (PDB #6V3G) is shown above in the sequences. The HRM motif (*CKACH*), methionine residues, and important residues for cytochrome c function are indicated in red. Despite variations in length (100–200 aa) and amino acid composition, these regions of the different BK channels share functional important residues.

This evidence suggests that the linker region may adopt a cytochrome c-like structure (known as the cytochrome c fold) within the C-terminal region of the BK channel, potentially stabilized by the presence of heme as a ligand. The RCK1-RCK2 linker region shows poor conservation across BK channels from different organisms, displaying variations in both length and amino acid composition compared to other regions of the BK channel. Notably, a primary sequence alignment of the region between the RCK1 and RCK2 domains reveals that crucial residues found in cytochrome c proteins—such as the HRM motif and the putative heme-coordinating methionine residue—along with functionally important positively charged residues, are highly conserved in this area (see Figure 4).

The evolutionary conservation of cytochrome c elements in the linker region of large-conductance potassium (BK) channels suggests that these elements play a crucial role in the physiological functioning of the channel. It is important to note that, while cytochrome c domains have been identified in other proteins, this study is the first to demonstrate cytochrome c-like features within an ion channel.

The Slo family of K^+^ channels includes three known members that are gated by voltage and are modulated differently: Slo-1 is regulated by Ca^2+^, Slo-2 is modulated by Cl^−^ and Ca^2+^, and Slo-3 is influenced by pH. A primary sequence alignment among the Slo family channels reveals that Slo-2 channels do not contain a conserved *CXXCH* motif. In contrast, the HRM CXXCH motif is present in Slo-1 (*^612^CKACH^616^*) and Slo-3 (*^612^CKSNH^616^*), and methionine residues are also conserved across these channels (Figure S1).

### 2.2. The Human BK Channel Confers Peroxidase Activity

Cytochrome c, one of the most intensively studied multifunctional proteins, and one of the first to be crystallized, is primarily known for its role in the respiratory chain [31].

Cytochrome c is found in various proteins and serves as an essential component in the electron transport chain, functioning as both an entry and an exit point for electrons during the enzyme’s catalytic cycle. Its primary role is to act as an electron carrier, transferring electrons from complex III to complex IV within the mitochondrial respiratory chain. The iron atom in the heme group alternates between two oxidation states—oxidized (Fe^3+^) and reduced (Fe^2+^)—as it facilitates the transport of electrons towards oxygen.

Recent studies on cell apoptosis have underscored the critical role of cytochrome c in programmed cell death. When an apoptotic signal is received, cytochrome c is released from the mitochondria into the cytoplasm. In the cytosol, it binds to APAF-1, which activates pro-caspase 9 and triggers an enzymatic cascade culminating in cell death. The release of cytochrome c and its involvement in apoptosis are regulated by multiple mechanisms closely tied to its catalytic and peroxidase activities [38,39,40].

We hypothesize that the human BK channel has peroxidase activity attributed to the RCK1-RCK2 linker region, which is proposed to function similarly to a cytochrome c-like domain (see Figure 1, Figure 2 and Figure 3). We investigate the peroxidase activity of both the C-terminal domain (Gating Ring) of the human BK channel and the full BK channel itself.

### 2.3. Peroxidase Activity of the Human BK Channel C-Terminal Domain

To investigate enzymatic activity, we expressed and purified the wild-type (WT), C615S/H616R, and M691A mutants of the human BK C-terminal domain (Figure S2A). This domain self-assembles in solution into a physiologically relevant tetrameric structure known as the gating ring. The mutations in the heme-coordinated site did not appear to affect the overall secondary structure of the gating ring, as indicated by the Circular Dichroism (CD) spectroscopy analysis of these channel proteins (Figure S2B) and Table 1.

The reaction catalyzed by many peroxidases may be generalized as:

ROOR + electron donor (2e^−^) + 2H^+^ → ROH + H_2_O

where ROOH can be hydrogen peroxide (H_2_O_2_) or an organic hydroperoxide.

A suitable electron donor is 2,2′-azino-bis(3-ethylbenzothiazoline-6-sulphonate) (ABTS), which can be used as a spectrophotometric reporter of peroxidase activity. The radical produced upon the oxidation of ABTS (ABTS^+•^) has maximal absorbance at ~415 nm, whereas the reduced form of ABTS absorbs maximally at ~340 nm. Thus, the time course of ABTS oxidation is ideal for the detection of peroxidase activity and the quantification of its reaction kinetics.

To spectroscopically probe for the enzymatic activity of the purified gating ring, we used the chromophore ABTS as the oxidizable substrate by H_2_O_2_ (see Material and Methods).

**Figure 5:**
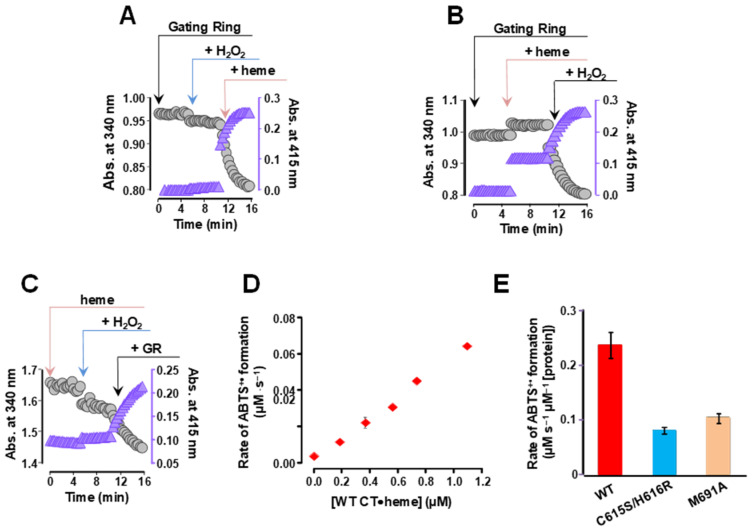
The formation of a gating ring/heme complex is essential for peroxidase activity. (**A**) Time course of ABTS oxidation in the presence of the gating ring alone (0.1 μM; 0–250 s), after the addition of H_2_O_2_ (500 μM at 250 s) and heme (2 μM at 600 s). (**B**) as in (**A**), with a different sequence of reagent additions: to gating ring + ABTS mixture was added heme at 250 s, and then added H_2_O_2_ 600 s. (**C**) Heme + ABTS mixture was supplemented with H_2_O_2_ at 250 s, and gating ring at 600 s. Note that the gating ring addition dramatically accelerated the peroxidase reaction, as in (**A**,**B**). (**D**) The initial rate of ABTS oxidation (v) was measured in increasing concentrations of the gating ring and plotted against [GR]. Note that the initial rate of ABTS^+•^ formation was linear concerning the concentration of the gating ring. (**E**) Quantification of peroxidase reaction rate from the data obtained from time course of ABTS oxidation in the presence WT, C615S/H616R and M691A at near saturation [heme], normalized by the estimated [CT•heme].

The time course for the oxidation of ABTS in the gating ring/heme complex reaction mixture is illustrated in Figure 5A–C. These experiments demonstrate that the BK channel intracellular gating ring exhibits catalytic properties, specifically, peroxidase activity. Both the gating ring protein and the heme prosthetic group are required for the peroxidase activity observed in this assay (Figure 5A–C).

We investigated the dependence of ABTS oxidation on the concentration of the BK gating ring protein complexed with heme ([CT●heme]), which was determined by the slope of the first 60 s of the reaction. As shown in Figure 5D, the initial rate of ABTS^+•^ formation linearly correlated with [CT●heme] up to 1.14 μM. We have also evaluated the role of heme-coordinating sites, the HRM (*^612^CKACH^616^*) in the peroxidase activity of the gating ring, and also the full BK channel, comparing the activities of the WT with and C615S/H616R and M691A gating ring mutants, respectively. We found that these mutations reduced the peroxidase activity of the gating ring, (Figure 5E). This result demonstrates that the peroxidase activity of the BK channel’s gating ring requires an intact heme-binding site.

**Figure 6:**
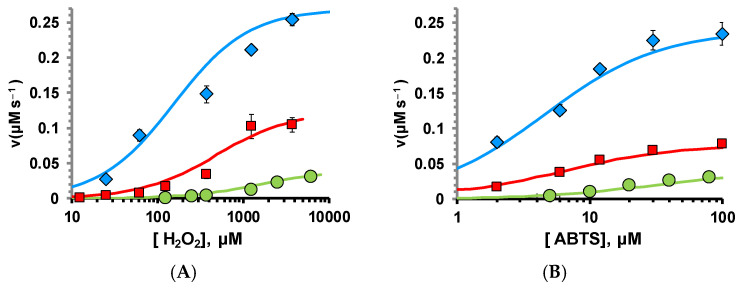
The BK gating ring peroxidase activity is augmented by STREX insertion. (**A**) Peroxidase reaction kinetic parameters of CytC (green circles), GR (red squares) and GR-STREX (cyan diamonds) were determined by varying [H_2_O_2_], while ABTS was used at a fixed concentration (50 μM) and (**B**) vice versa (varying [ABTS] while [H_2_O_2_] = 0.7 mM) in the presence of 2 µM. The data for each protein were fit simultaneously using a two-substrate Michaelis–Menten kinetic model. Parameters are shown in Table 2.

To address the mechanism of the enzymatic degradation of H_2_O_2_ by the gating ring, we compared the kinetic parameters of human mitochondrial cytochrome C (hCytC) and the BK gating ring activity using a two-substrate Michaelis–Menten kinetic model (Figure 6, Table 2). Although the intrinsic catalytic rates (*k*_cat_) of the gating ring and hCytC are very similar, the human BK channel C-terminal exhibits higher apparent affinity for H_2_O_2_ and ABTS substrates than human cytochrome c: ~11-fold higher for H_2_O_2_ and ~8-fold higher for ABTS.

### 2.4. STREX Significantly Augmented CTD Peroxidase Activity

BK channels exhibit rich splice variation in tissues. One of the best-characterized alternative splice variants includes the stress-axis-regulated exon (STREX). Currents in inside-out patches pulled with STREX activated at voltages −20 mV negative to those with zero [41,42]. The inclusion of STREX results in a leftward shift in the conductance voltage curve with a Vh at 10 µM −22.33 ± 0.30 mV, compared with zero, which shows 20.5 ± 1.44 mV [43]. The insertion of STREX also alters properties, such as BK channel properties, including BK channel modulation by phosphorylation [44] and palmitoylation [45]. As STREX (58 aa) is inserted in the RCK1-RCK2 linker (Figure S3), it could alter the functional activities of the BK CTD. Indeed, the inclusion of STREX significantly augmented CTD peroxidase activity. Assuming the same heme-binding properties, the peroxidase *k*_cat_ value was increased by ~2-fold, while the apparent substrate affinity was increased by ~3-fold for both H_2_O_2_ and ABTS.

### 2.5. The Full Human BK Channel Exhibits Peroxidase Activity

To evaluate the peroxidase activity of the full BK channel in HEK293 cell lysates, we assessed peroxidase activity using the Amplex Red assay, which measures the resorufin fluorescence in the result oxidation of Amplex Red. Amplex Red reagent is a colorless substrate that reacts with hydrogen peroxide (H_2_O_2_) to produce highly fluorescent resorufin (emission maxima around 580 nm) in the presence of the peroxidase. Peroxidase activity assay performed in HEK293 cell lysates. HEK293 cells were transfected to yellow fluorescent protein with (YFP) or YFP was fused to the C-terminus of WT or C615S/H161R mutant human BK channel constructs (Figure S4). YFP fluorescence signal fused to the BK channel proteins allows the expression level with the emission peak 527 nm to be quantified, at an excitation of 500 nm. To determine the concentrations of expressed YFP, as well as YFP fused to the BK WT and BK C615S/H616R proteins in HEK lysates, we utilized the fluorescence emission intensity of YFP at 527 nm (with an excitation wavelength of 500 nm). After measuring the total protein concentration in the HEK cell lysates, we used the emission intensity at 527 nm to standardize for the equal amount of expressed YFP, YFP fused to BK WT, and BK C615S/H616R proteins in the Amplex Red assay.

**Figure 7:**
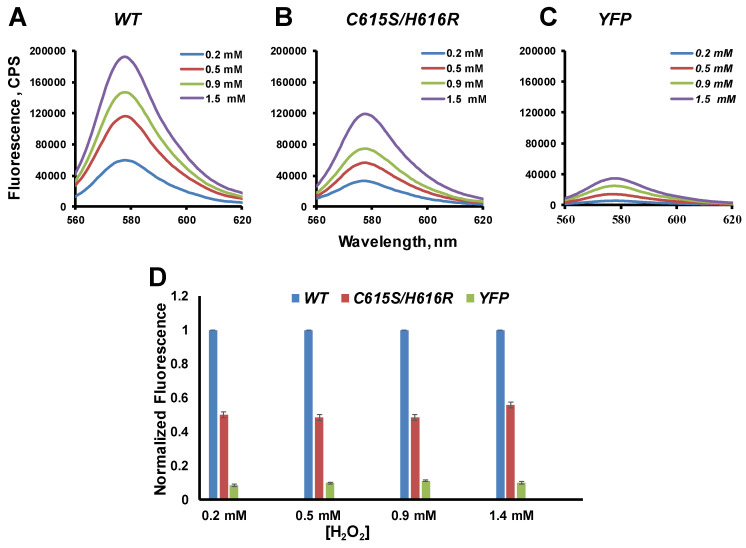
The full human BK channel exhibits peroxidase activity. (**A**) Emission fluorescence spectra of the resorufin, the oxidation product of Amplex Red (λext 540 nm), obtained at increasing concentrations of H_2_O_2_. Lanes, (**A**–**C**) HEK293 cells lysates expressed with WT BK-YFP, C615S/H616R BK-YFP and YFP alone, respectively. (**D**) Normalized fluorescence intensity at emission peak at 580 nm obtained from HEK293 cells lysates, which expressed with YFP alone, WT BK-YFP, and C615S/H616R BK-YFP at different concentrations of H_2_O_2_.

The intensity of resorufin fluorescence formed in the in BK-transfected cells lysates were compared to that in cells expressing YFP alone and cells transfected with a BK HRM mutant with impaired heme-binding ability (C615S/H616R). This experiment revealed a high resorufin fluorescence intensity in BK channel-expressed cells compared to cells expressing only YFP (Figure 7). These data demonstrated that the full BK channel can produce formation resorufin due to its peroxidase activity. We found that the C615S/H616R mutations reduced the fluorescence intensity of resorufin, indicating that heme-coordinating sites are critical for the peroxidase activity of the BK channel (Figure 7B).

## 3. Discussion

### 3.1. Cytochrome-C-like Structure Within BK Channel Gating Ring

Heme serves as a novel regulator of various physiological processes and is essential for living organisms [46,47]. Recent studies have revealed that heme modulates the activity of ion channels [3,11,24,26,31]. However, the molecular mechanisms behind heme-dependent regulatory control in ion channels remain unclear. The interaction sites where ion channels bind with heme are not well characterized and may vary across different ion channels. Currently, there is no evidence that heme binds directly to ion channels, and the specific heme-binding sites on ion channel proteins have not been identified. Attempts to obtain a co-crystal structure of heme-bound ion channel proteins have been unsuccessful [48].

In this study, we are addressing the molecular-basis heme-dependent regulation activity of the BK channel. We found that the heme-coordinated region of the human BK channel located within the ~120 residues linker connecting two modules, RCK1 and RCK2 domains share similarities with cytochrome c family proteins (Figure 2C). Like cytochrome c fold proteins, the structure of N- and C-terminals of this region is α helices, and in addition to the HRM (*CXXCH*), M691 is also conserved, which aligns with the second axial ligand to the heme iron Met residues of cytochrome c family proteins. Cytochrome c achieves several diverse roles in cells. Its involvement in electron-transfer reactions between III and IV complexes, happening via transient protein–protein interfaces, is dependent on electron tunneling and conformational dynamic [49]. This performance is driven by the cyt c’s hexa-coordinate structure with His18 and Met80 as the two axial ligands [40]. In contrast, another critical role of cytochrome c in early apoptosis requires the disruption of the Met80-heme iron bond, which results in the partial unfolding of the protein, and increasing peroxidase activity of the protein [40,50].

To gain more insight into the structure of the RCK1-RCK2 linker region, we compared in detail its primary sequences with the sequences of this region (Figure 2A). This analysis revealed that critical functional important positively charged residues (K7, K22, K25, K39, K72, K73, and K86) for Apaf-1 and cardiolipin interaction align well with BK residues K606, K627, K630, K643, R631, K684, K685, and K698 (Figure 1A).

We hypothesized that the linker region between RCK1 and RCK2 may form a structure similar to cytochrome c in the presence of heme, with HMR and M691 serving as axial ligands for the heme iron.

The following experiments are designed to test this hypothesis and to understand the role of the cytochrome c-like structure in the functioning of the BK channel. We focused on the peroxidase activity of the BK channel, as this is an important functional property of cytochrome c. To pharmacologically eliminate the ion-conducting properties of the channels and concentrate our investigation on the enzymatic activity of the gating ring, we supplemented the cell medium with 1 µM of a highly selective and potent BK channel blocker, which has an effective Kd of approximately 10 nM [51]. This approach ensured that the BK channel conductance was fully blocked while leaving the native ionic conductance unaffected. The strong blockade of the BK channel indicates that the observed peroxidase activity is mediated by its non-conducting properties.

We have demonstrated that the linker region between RCK1-RCK2 domains equips the full BK channel with peroxidase functionality (Figure 4, Figure 5 and Figure 6). We tested the contribution of the HRM and M691 residue for heme coordination by this catalytic activity of the gating ring, as well as the full channel (Figure 6). The mutations H616R and C615S in HRM and M691A mutations reduced the enzymatic performance of the channel. These results reinforce the view that a cytochrome-c-like structure that associates heme via the HRM and a second axial ligand (Met-691) is an integral part of BK channels. These results suggest that the inhibitory effect of heme on the BK channel gating is mediated by heme binding to the HRM [22], as well as to M691.

We observed an interesting appositive effect when the STREX variant was inserted into the linker region (Figure 6). The stress-regulated exon (STREX) of the BK channel is the most extensively studied splice variant and is highly conserved among vertebrates. This cysteine-rich region leads to significant changes in the BK channel’s characteristics, including slower deactivation and a notable leftward shift in the voltage required for half-maximal activation [43,52]. The STREX insert interacts with multiple signaling pathways, making it sensitive to inhibition by acute hypoxia and oxidation. This functionality relies on the highly conserved cysteine residues found in the Cys-Ser-Cys (CSC) motif [53].

STREX is positioned within the linker region adjacent to the HRM. Further investigation is needed to determine how STREX may enhance peroxidase activity and whether the CSC motif plays a role in this process. One potential explanation is that the inclusion of STREX could influence the conformation of the disordered region between RCK1 and RCK2. Additional research may provide more insights into the physiological relevance of the peroxidase activity of the BK channel.

### 3.2. The Novel Multifunctional Module Within BK Channels

Our data obtained based on bioinformatics and biochemical approaches indicate that the linker region between RCK-RCK2 domains of the BK channel-forming α subunit includes cytochrome c family-like structural elements. These findings proposed that heme binding may be from cytochrome c fold inside the BK channel (Figure 2B). This region is largely unresolved in reported crystal structures due to its unordered structure [15,16,17,19]. This part of the channel consists of only a few secondary structure elements, α helices (Figure 2C). The heme-driven formation of compact cytochrome c fold structure will be associated with a considerably disorder-to-order conformational transition upon heme binding. These structural rearrangements within the gating ring may lie under the structural basis of the heme-dependent modulation activity of the BK channels. Finding a novel heme-sensitive site in the extracellular space of mitoBK suggests that heme may affect BK channel activity through multiple mechanisms [20].

Beyond that, this region appears responsible for heme-driven regulation of BK channel activity, and it may also provide novel performances underlying important physiological functions of these exceptional ion channel proteins due to similar activities as the “extreme multifunctional” protein cytochrome c [38].

During the past decade, a fascinating body of experimental investigations has provided evidence that voltage-gated ion channels may do more than their well-described function in regulating electrical excitability, such as participating in intracellular signaling pathways, in cell behavior. The BK channel has recently been shown to play a protective function against ischemia–reperfusion injury both in vitro and in animal models, although the exact mechanism of this protection remains unknown [54,55]. It was demonstrated that in the absence of BK, post-anoxic reactive oxygen species production was elevated in mouse cardiomyocyte mitochondria [56]. While the contribution of the mitochondrial BK channel conductance to this effect is unknown, their ability to break down H_2_O_2_ may play a part in the protective mechanism of ischemic pre-conditioning.

Here, we report that the human BK channel possesses peroxidase activity as a function of the cytochrome c domain, reinforcing the view that ion channels are more than ion-conducting structures. Notably, since all Amplex Red assay experiments were conducted with BK channels (using 1 μM paxillin) [51], the results emphasize that the peroxidase activity of the BK channel does not rely on its ion-conducting properties. Furthermore, these findings closely replicate the data from our biochemical experiments, which demonstrate a reduction in peroxidase activity in the gate ring containing the C615S/H616R mutations (Figure 5). To our knowledge, this is the first demonstration of enzymatic activity exhibited by BK channels, and one of the few generally demonstrated in ion channels. Although the full understanding of the physiological functions and implications of this enzymatic activity will require extensive future investigations.

## 4. Materials and Methods

### 4.1. Expression and Purification of BK-Terminal Proteins

The intracellular C-terminal domain (CTD) (*^322^IIE…ALK^1005^*) WT, HRM (C615S/H616R) and M691A mutant, as well as CTD including STREX CTD constituent RCK1 (*^322^IIE…HDP^667^*) and RCK2 (*^665^HDP…ALK^1005^*) domains, were expressed and purified from *E. coli* cells as described previously [10,57,58]. Protein concentrations were estimated using Micro Lowry, Peterson’s Modification (Merck, Rahway NJ, USA).

The far-UV spectra of purified whole GR, and its mutant proteins were recorded between 190 and 260 nm by using a quartz cell of 0.1 cm path length. Spectra were collected at an average of 7–9 scans. Secondary structure fractions were calculated using the SMP56 or CLSTR reference set of the CDPro software package [58,59]. NRMSD (normalized root mean-square deviation) was used as a measure of the goodness of fit to the experimental spectrum.

### 4.2. ABTS Assay

The peroxidase activity of the WT and mutant human BK channel gating ring was investigated using a colorimetric assay with 2,2′-azino-bis-(3-ethylbenzothiazoline-6-sulfonate) (ABTS) as an electron donor. Reduced ABTS has a characteristic peak at 340 nm; when the peroxidase-catalyzed reduction of H_2_O_2_ to water is coupled to the oxidation of ABTS, an absorbance peak at 415 nm is formed. The reaction mixture (100 μL quartz cuvette) included 20–50 μM ABTS, 10–500 μM H_2_O_2_, and 0.1–2 μM BK C-terminus (WT or mutants) complexed with 0.4–8 μM heme. Peroxidase activity was assayed in experiments with a heme concentration of 2 μM.

Michaelis–Menten catalytic kinetic parameters were estimated at varying concentrations of substrates (ABTS or H_2_O_2_) using a two-substrate Michaelis–Menten kinetic model:

$$v=\frac{\left[\mathrm{Enzyme}\right]}{\left(\frac{1}{k_{\mathrm{cat}}}+\frac{K_{m}^{H_{2}O_{2}}}{k_{\mathrm{cat}}\cdot \left[H_{2}O_{2}\right]}+\frac{K_{m}^{\mathrm{ABTS}}}{k_{\mathrm{cat}}\cdot \left[\mathrm{ABTS}\right]}\right)}$$

in which *v* is the initial rate; *k*_cat_ is the intrinsic catalytic activity; *K*_m_^H_2_O_2_^ and *K*_m_^ABTS^ are the equilibrium binding constants for H_2_O_2_ and ABTS, respectively; [Enzyme] is the enzyme concentration, i.e., CytC or the BK CT/heme complex.

### 4.3. Peroxidase Activity Assay in Cell Lysates

HEK293 cells (ATCC: CRL-1573, kind gifts from Dr. Liqia Toro (UCLA)) were transfected with YFP, or YFP was fused to the C-terminus of WT or C615S/H161R mutant BK channel constructs using lipofectamine 2000). Cells were grown in the presence of the specific BK channel blocker 1 μM paxilline (PAX). After 72 h, collected cells were lysed in a buffer (50 mM Tris HCI, 2 mM EDTA, pH 7.4) in the presence of a protease inhibitor cocktail (Roche) and 1 mM PMFS using sonication (5–10 s) and then centrifuged at 3000× *g* for 5 min to clarify the lysates. The expression level of channel proteins was evaluated using the fluorescence intensity of the YFP signal (λext 500 nm/λem 527 nm). Protein concentration was measured using the DC protein assay kit (Bio-Rad Laboratories, Hercules, CA, USA). Peroxidase activity in the cell lysates was determined using the Amplex Red assay. The assay solution contained 50–100 µg protein samples, 5 µM Amplex Red, and various concentrations of H_2_O_2_ in a 200 µL lysis buffer, and fluorescence of the resorufin, oxidation product of Amplex Red, was detected at emission 580 nm (λext 540 nm).

## Figures and Tables

**Table 1:** Secondary structure composition of the WT, mutants C615S/H616R and M691A of the gating ring. The relative secondary structure composition of the BK gating ring was calculated using the CONTIN/LL program and SMP56 reference set of the CDPro software package from circular dichroism spectra in Supplementary Figure S2B.

	WT GR [10]	(C615S/H616R) GR in This Study	M691A GR in This Study
α-helix (%)	28.6	27.8	28.0
nα-helix	21.9	21.5	21.3
β-strand (%)	21.8	22.1	21.1
nβ-strand	31.2	30.9	30.5
Turn + Unordered (%)	49.7	50.2	50.8
NRMSD	0.018	0.015	0.017

**Table 2:** Two-substrate, Michaelis–Menten kinetic parameters for H_2_O_2_ oxidation of ABTS by heme-bound human BK channel gating ring with or without the STREX insert, and CytC. Experiments are shown in Figure 6.

Protein	*K*_m_^H2O2^ (mM)	*K*_m_^ABTS^ (mM)	*k*_cat_
GR	0.512 ± 0.1	0.012 ± 0.001	0.14 ± 0.02
GR/STREX	0.15 ± 0.01	0.0043 ± 0.0009	0.29 ± 0.018
CytC	5.49 ± 0.34	0.1 ± 0.002	0.13 ± 0.0165

## Data Availability

The original contributions presented in this study are included in the article/Supplementary Materials. Further inquiries can be directed to the corresponding author(s).

## Supplementary Materials

The supporting information can be downloaded at: https://www.mdpi.com/article/10.3390/ijms26157053/s1.
