# Supplementary material for: Cytochrome C-like Domain Within the Human BK Channel
Source: Int J Mol Sci. 2025 Jul 22;26(15):7053. doi: 10.3390/ijms26157053 (PMC12346439; doi:10.3390/ijms26157053)
Supplement: Supplementary file 1 [file ijms-26-07053-s001.zip › ijms-3474083-supplementary.pptx]

## Slide 1
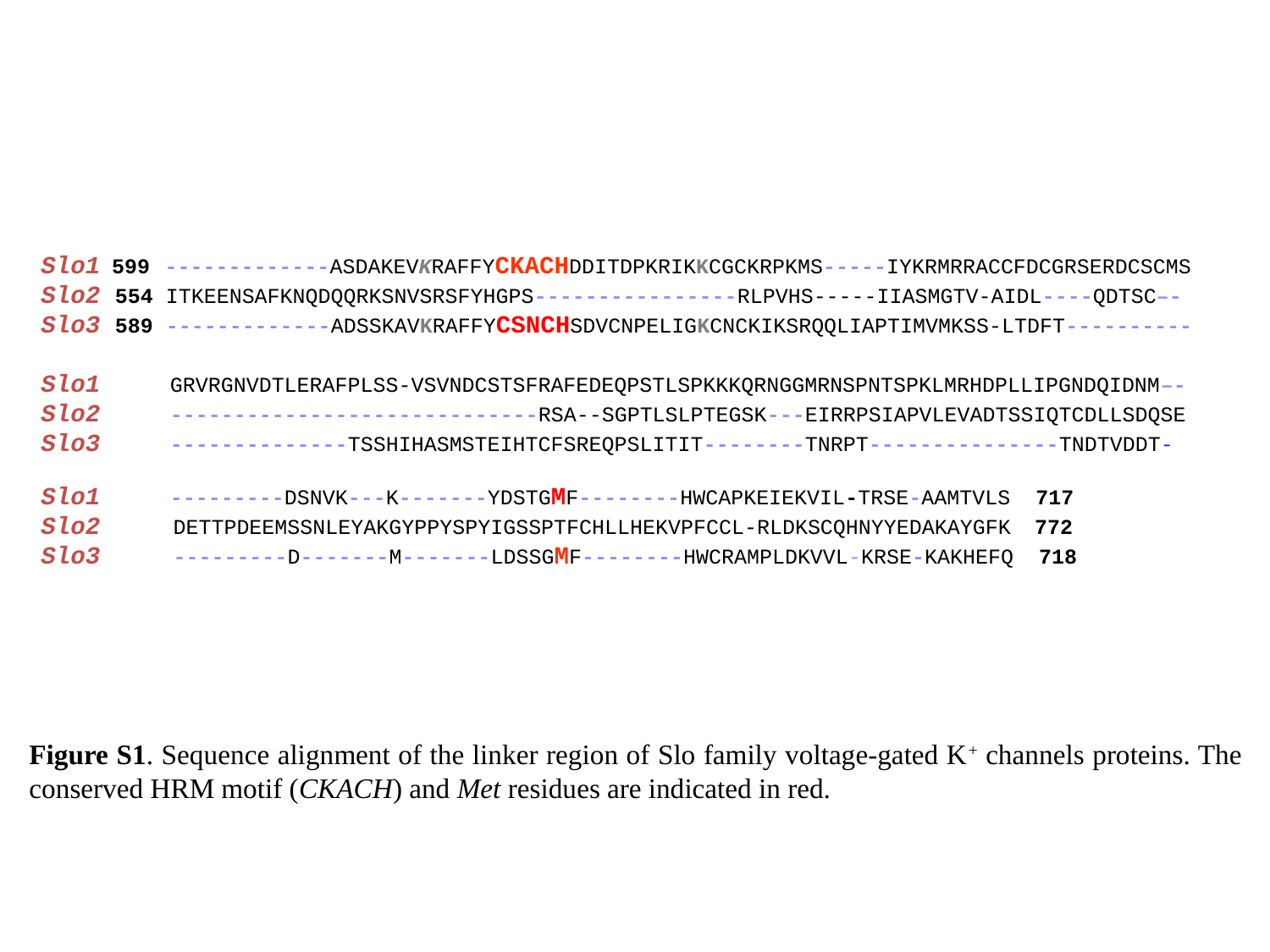

Slo1 599 -------------ASDAKEVKRAFFYCKACHDDITDPKRIKKCGCKRPKMS-----IYKRMRRACCFDCGRSERDCSCMS
Slo2 554 ITKEENSAFKNQDQQRKSNVSRSFYHGPS----------------RLPVHS-----IIASMGTV-AIDL----QDTSC–-
Slo3 589 -------------ADSSKAVKRAFFYCSNCHSDVCNPELIGKCNCKIKSRQQLIAPTIMVMKSS-LTDFT----------
Slo1 GRVRGNVDTLERAFPLSS-VSVNDCSTSFRAFEDEQPSTLSPKKKQRNGGMRNSPNTSPKLMRHDPLLIPGNDQIDNM–-
Slo2 -----------------------------RSA--SGPTLSLPTEGSK---EIRRPSIAPVLEVADTSSIQTCDLLSDQSE
Slo3 --------------TSSHIHASMSTEIHTCFSREQPSLITIT--------TNRPT---------------TNDTVDDT-
Slo1 ---------DSNVK---K-------YDSTGMF--------HWCAPKEIEKVIL-TRSE-AAMTVLS 717
Slo2 DETTPDEEMSSNLEYAKGYPPYSPYIGSSPTFCHLLHEKVPFCCL-RLDKSCQHNYYEDAKAYGFK 772
Slo3 ---------D-------M-------LDSSGMF--------HWCRAMPLDKVVL-KRSE-KAKHEFQ 718
Figure S1. Sequence alignment of the linker region of Slo family voltage-gated K+ channels proteins. The conserved HRM motif (CKACH) and Met residues are indicated in red.

## Slide 2
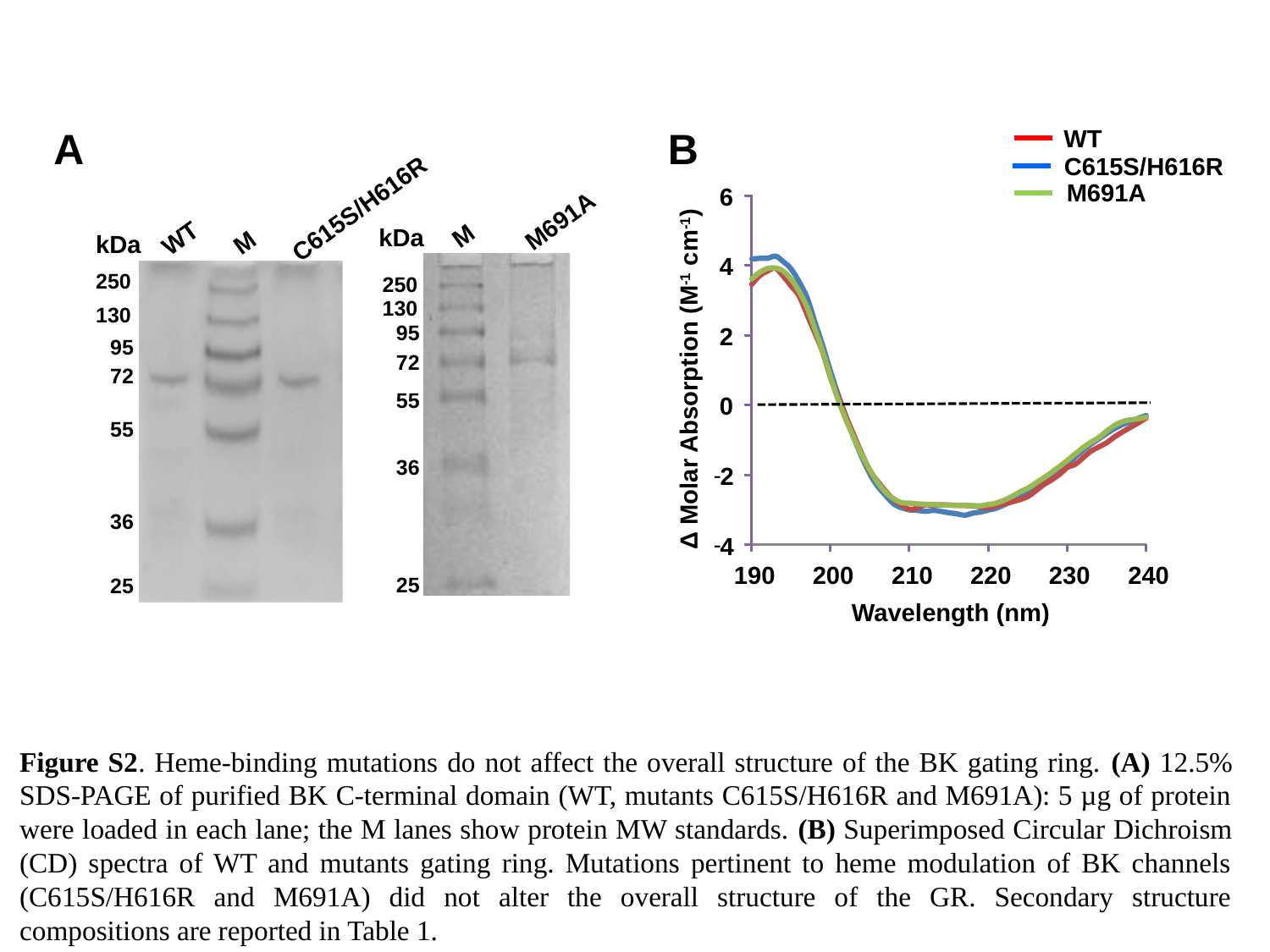

A
B
WT
C615S/H616R
M691A
6
4
2
0
-
2
-
4
190
200
210
220
230
240
C615S/H616R
M691A
M
kDa
WT
M
kDa
250
250
130
130
95
95
72
Δ Molar Absorption (M-1 cm-1)
72
55
55
36
36
25
25
Wavelength (nm)
Figure S2. Heme-binding mutations do not affect the overall structure of the BK gating ring. (A) 12.5% SDS-PAGE of purified BK C-terminal domain (WT, mutants C615S/H616R and M691A): 5 µg of protein were loaded in each lane; the M lanes show protein MW standards. (B) Superimposed Circular Dichroism (CD) spectra of WT and mutants gating ring. Mutations pertinent to heme modulation of BK channels (C615S/H616R and M691A) did not alter the overall structure of the GR. Secondary structure compositions are reported in Table 1.

## Slide 3
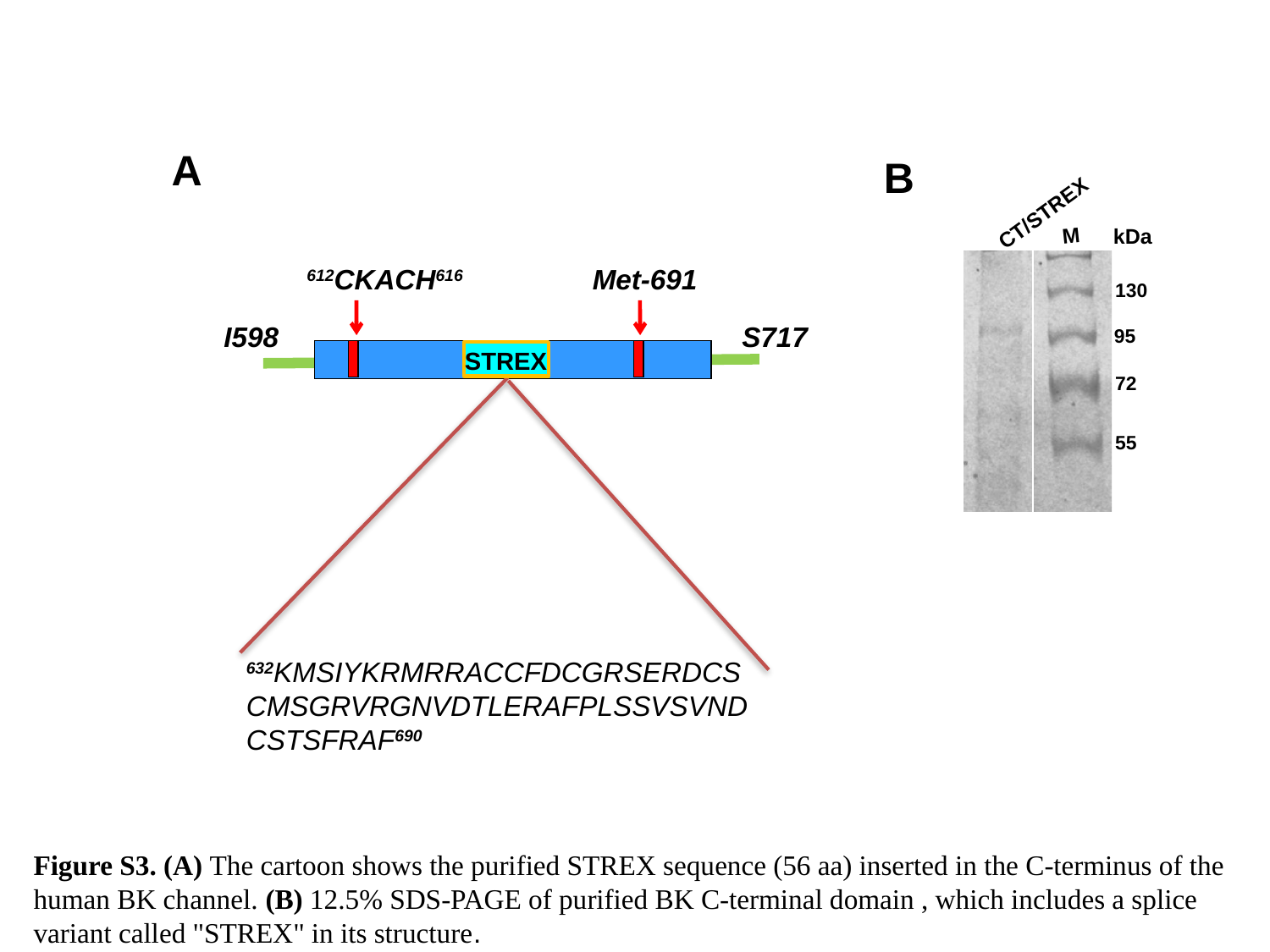

A
B
CT/STREX
M
kDa
612CKACH616
Met-691
130
RK2
I598
S717
95
STREX
72
55
632KMSIYKRMRRACCFDCGRSERDCSCMSGRVRGNVDTLERAFPLSSVSVNDCSTSFRAF690
Figure S3. (A) The cartoon shows the purified STREX sequence (56 aa) inserted in the C-terminus of the human BK channel. (B) 12.5% SDS-PAGE of purified BK C-terminal domain , which includes a splice variant called "STREX" in its structure.

## Slide 4
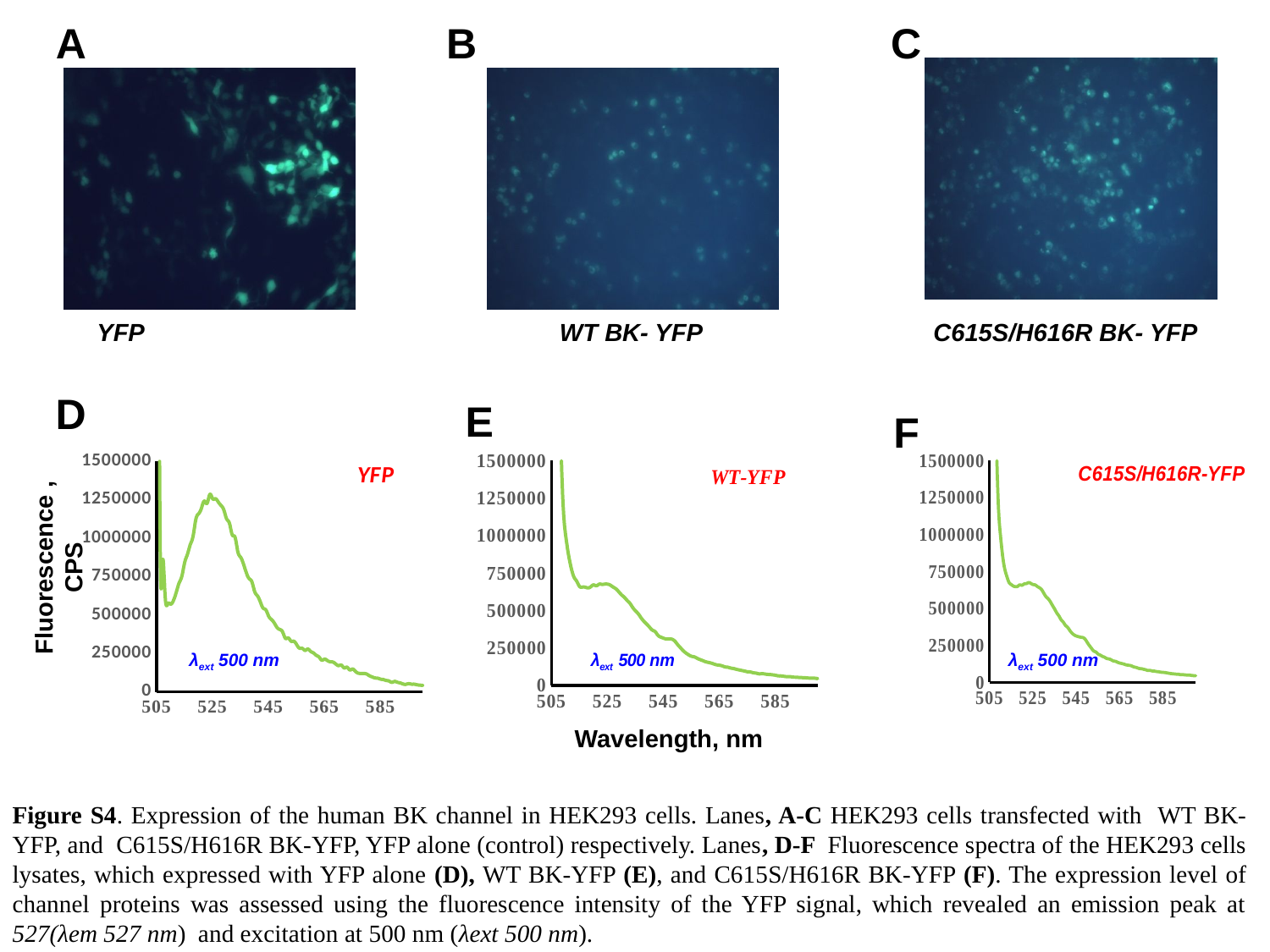

A
B
C
 WT BK- YFP
C615S/H616R BK- YFP
 YFP
D
E
F
### Chart:
| Category | YFP |
|---|---|
### Chart:
| Category | WT-YFP |
|---|---|
### Chart:
| Category | C615S/H616R-YFP |
|---|---|Fluorescence , CPS
λext 500 nm
λext 500 nm
Wavelength, nm
Figure S4. Expression of the human BK channel in HEK293 cells. Lanes, A-C HEK293 cells transfected with WT BK-YFP, and C615S/H616R BK-YFP, YFP alone (control) respectively. Lanes, D-F Fluorescence spectra of the HEK293 cells lysates, which expressed with YFP alone (D), WT BK-YFP (E), and C615S/H616R BK-YFP (F). The expression level of channel proteins was assessed using the fluorescence intensity of the YFP signal, which revealed an emission peak at 527(λem 527 nm)  and excitation at 500 nm (λext 500 nm).
